# CRISPR/Cas9-mediated mutagenesis of the susceptibility gene *OsHPP04* in rice confers enhanced resistance to rice root-knot nematode

**DOI:** 10.3389/fpls.2023.1134653

**Published:** 2023-03-14

**Authors:** Qiuling Huang, Borong Lin, Yuqing Cao, Yu Zhang, Handa Song, Chunhui Huang, Tianling Sun, Changwen Long, Jinling Liao, Kan Zhuo

**Affiliations:** ^1^ Laboratory of Plant Nematology, College of Plant Protection, South China Agricultural University, Guangzhou, China; ^2^ Guangdong Province Key Laboratory of Microbial Signals and Disease Control, South China Agricultural University, Guangzhou, China

**Keywords:** rice, susceptibility gene, OsHPP04, disease resistance, host immune, *Meloidogyne graminicola*, CRISPR/Cas9

## Abstract

CRISPR crops carrying a mutation in susceptibility (*S*) genes provide an effective strategy for controlling plant disease, because they could be ‘transgene-free’ and commonly have more broad-spectrum and durable type of resistance. Despite their importance, CRISPR/Cas9-mediated editing of *S* genes for engineering resistance to plant-parasitic nematode (PPN) disease has not been reported. In this study, we employed the CRISPR/Cas9 system to specifically induce targeted mutagenesis of the *S* gene rice copper metallochaperone heavy metal-associated plant protein 04 (*OsHPP04*), and successfully obtained genetically stable homozygous rice mutants with or without transgenic elements. These mutants confer enhanced resistance to the rice root-knot nematode (*Meloidogyne graminicola*), a major plant pathogenic nematode in rice agriculture. Moreover, the plant immune responses triggered by flg22, including reactive oxygen species burst, defence-related genes expression and callose deposition, were enhanced in the ‘transgene-free’ homozygous mutants. Analysis of rice growth and agronomic traits of two independent mutants showed that there are no obvious differences between wild-type plants and mutants. These findings suggest that *OsHPP04* may be an *S* gene as a negative regulator of host immunity and genetic modification of *S* genes through the CRISPR/Cas9 technology can be used as a powerful tool to generate PPN resistant plant varieties.

## Introduction

Rice (*Oryza sativa* L.) is the most important staple food worldwide, sustaining more than 50% of the world’s population, particularly in Asia. However, the production and quality of rice are substantially affected by various plant pathogens. *Meloidogyne graminicola*, a root-knot nematode (RKN) species, is one of the most prevalent pathogens in rice cultivation system and responsible for yield losses from 17% to 32% (Kyndt et al., 2014; Mantelin et al., 2017). Most rice cultivars are vulnerable to *M. graminicola*, and the field population of *M. graminicola* is increasing dramatically due to changes in farming practices (De Waele and Elsen, 2007).

The most effective and eco-friendly approach for managing RKN disease is to breed and deploy resistant cultivars (Kaloshian and Teixeira, 2019). Conventional breeding relies on the deployment of dominant resistance (*R*) genes, which typically encode nucleotide-binding leucine-rich repeat proteins (NLRs) that directly or indirectly recognize pathogenic effector proteins and trigger host innate immunity (Jones and Dangl, 2006; St Clair, 2010). Currently a few highly resistant varieties have been discovered within the Asian rice germplasm bank, such as Sri Lanka’s *indica* rice LD24, Thailand’s *aus* rice Khao Pahk Maw (KPM), and Chinese *japonica* rice Zhonghua11 (ZH11) and Huaidao 5 (HD5) (Dimkpa et al., 2016; Hada et al., 2020; Feng et al., 2022). Moreover, recent studies suggested that *M. graminicola* resistance is governed by a major locus on chromosome 11 in *Oryza sativa* L. (Lahari et al., 2019), and Phan et al. (2018) proposed that a major resistance gene possibly involved in the ZH11 resistance to *M. graminicola* infection. However, the potential dominant *R* gene has not been cloned so far.

As an alternative, disease resistance can also be achieved by disruption of susceptibility (*S*) gene (Zaidi et al., 2018). *S* genes are plant genes that are induced and/or targeted by pathogens for host recognition, penetration, nutrient acquisition, proliferation, and spread or for the inhibition of the host immune signaling (van Schie and Takken, 2014; Zaidi et al., 2018). Resistance conferred by *S* gene inactivation is genetically recessive and durable. A classic example is the *S* gene *MILDEW RESISTANCE LOCUS O* (*MLO*) of barley. *MLO*-based resistance to powdery mildew has been effectively used in barley cultivation for about four decades in Europe (Acevedo-Garcia et al., 2014). With advances in genome editing technologies, especially the clustered regularly interspaced short palindromic repeats/CRISPR-associated protein 9 (CRISPR/Cas9) system, it has brought a breakthrough in breeding new resistant materials through *S* gene editing (Langner et al., 2018; Zaidi et al., 2018; Li et al., 2020). However, the strategy used by CRISPR/Cas9-mediated editing of *S* genes for engineering resistance plants to plant-parasitic nematode (PPN) disease has not been reported so far.

In our previous study, the rice gene *OsHPP04* was demonstrated to have a CysXXCys motif within a βαββαβ-fold heavy metal-associated domain (HMA), in which the Cys residues may bind Cu. The further yeast expression assay confirmed its Cu binding specificity, indicating that OsHPP04 is a copper metallochaperone heavy metal-associated plant protein (HPP). HPPs are common in plants, which are usually involved in different biological processes, such as heavy metal homeostasis, transport and detoxification, responses to abiotic stresses and plant-pathogen interactions. The expression of *OsHPP04* was found to be induced in rice galls and rice plants overexpressing *OsHPP04* exhibited an increased susceptibility to *M. graminicola* (Song et al., 2021), implying that *OsHPP04* might be an *S* gene for *M. graminicola.* In this research, we generated loss-of-function mutants of the rice *S* gene *OsHPP04* by CRISPR/Cas9-mediated gene editing. We demonstrated that the *OsHPP04*-knockout rice plants had an enhanced resistance to *M. graminicola* compared to wild-type (WT) rice. The flg22-induced pathogen-associated molecular pattern (PAMP)-triggered immunity (PTI) responses, including the production of reactive oxygen species (ROS), expression level of defence-related genes and deposition of cell wall callose, were obviously increased in the *OsHPP04*-knockout lines compared with that in WT rice. Moreover, the mutant lines did not show any significant differences in major agronomic traits compared to the WT control. Taken together, our data indicated that *OsHPP04* could be an *S* gene as a negative regulator of host immunity and demonstrated the effectiveness of *S* gene editing by the CRISPR/Cas9 technology for generating PPN resistant plant resources.

## Materials and methods

### Nematode and plant materials

*Meloidogyne graminicola* was collected from rice in Hainan, China, then purified and cultured on rice (*Oryza sativa* cv. ‘Nipponbare’) in a greenhouse at 27°C, under a 16 h/8 h light/dark regime. Egg masses and pre-parasitic second stage juveniles (pre-J2s) were collected as previously described (Huang et al., 2020). Rice seeds, including WT and mutant lines, were germinated on B5 medium at 27°C with a light/dark photoperiod of 16 h/8 h, then were transferred and cultivated in sand and soil mixture (3:1) at 27°C in a greenhouse, under a light regime of (light: dark) 16: 8 h.

### CRISPR/Cas9 vector construction and rice transformation

Sequence-specific gRNAs were designed based on the web-based tool CRISPR-P (http://cbi.hzau.edu.cn/cgi-bin/CRISPR) (Liu et al., 2017). Two gRNAs of the target sites of the *OsHPP04* gene were selected according to their location in the gene, GC% content and putative off-targets. The binary pYLCRIPSR/Cas9 multiplex genome targeting vector system, including pYLCRISPR/Cas9Pubi-H, pYLgRNA-OsU6a-Lacz and pYLgRNA-OsU6b, were gifted by Professor Yaoguang Liu from South China Agricultural University. The sgRNA cassettes driven by OsU6a and OsU6b, respectively, were inserted into pYLCRISPR/Cas9Pubi-H according to the method described previously (Ma et al., 2015), to obtain the CRISPR/Cas9 knockout vector of *OsHPP04*. The oligos used to construct the CRISPR/Cas9 knockout vector and all other primers used in this study are listed in **Supplementary Table 1**.

The transgenic rice plants were generated as previously described (Li et al., 2022). The CRISPR/Cas9 knockout plasmid was transformed into *Agrobacterium tumefaciens* strain EHA105, and then infected the callus tissue induced from Nipponbare seeds. Transformants were selected by 50 mg/L hygromycin in Bon Chu’s N6 medium.

### Mutation detection and identification of ‘transgene-free’ homozygous mutant plants

All hygromycin resistant transgenic T_0_ rice plants were used for further analysis. Rice genomic DNA was extracted from leaves according to the CTAB method (Rowland and Nguyen, 1993). Transgenic T_0_ rice plants were confirmed by PCR using the Cas9-specific primers Cas9-F/Cas9-R. Subsequently, the DNA fragment across the *OsHPP04* target site was amplified using the specific primer pair OsHPP04-Pf/OsHPP04-Pr. The PCR amplicons were directly sequenced using the primer OsHPP04-cx-F. The sequencing chromatograms with superimposed peaks of bi-allelic and heterozygous mutations were decoded using the Degenerate Sequence Decoding method (http://skl.scau.edu.cn/dsdecode/) (Liu et al., 2015).

The identification of homozygous mutant plants was conducted from T_1_ generation plants as described above. To identify ‘transgene-free’ homozygous mutant plants, the T_1_ mutant plants were analyzed by PCR using Cas9-specific and sgRNA-specific primer pairs, i.e. Cas9p-F/R and sgRNA-F/R, and agarose gel electrophoresis. The 18S rRNA (AK059783) was used as a normalization control and amplified by the specific primer pair 18S rRNA-F/R (Chen et al., 2018).

### Off target analysis

The potential off-target sites of the two gRNAs of *OsHPP04* were identified using CRISPR-P (http://cbi.hzau.edu.cn/cgi-bin/CRISPR) against the reference genome (Liu et al., 2017). An approximately 500-bp DNA fragment covering each off-target site was amplified by PCR. The PCR products of six T_0_ plants were sequenced and compared with WT rice sequences.

### Nematode inoculation

The rice seedlings (including WT and homozygous mutant T_2_ lines) were cultured in B5 medium plates as mentioned above. The 10-day-old seedlings were transplanted to a 14 cm long PVC tube filled with a mixture of white sand and superabsorbent polymers (SAP), as previously described (Naalden et al., 2018). The plants were grown for 14 d in a greenhouse at 27°C with a light/dark photoperiod of 16 h/8 h, and watered with Hoagland solution as a source of nutrients every three days. Then fourteen-day-old rice plants were inoculated with 200 *M. graminicola* pre-J2s. At 12 day post inoculation (dpi), roots were collected, washed and stained by acid fuchsin, and the number of females was counted (Naalden et al., 2018). Each experiment was performed three independent times. Statistical differences between treatments and controls were calculated by Student’s *t* test.

### Immune assay

The ROS assay was performed using a luminol-based method, as previously described (Tian et al., 2020). In brief, approximately 1-cm basal stem of 10-day-old homozygous mutant and WT rice plants were dispatched on a 96-well plate and incubated in H_2_O for 4 h. Then the water was removed, and 100 μl of luminol-based reaction buffer, including 17 mM luminol (Sigma, USA, SKU No. 123072-2.5G), 1 mM horseradish peroxidase (Sigma, USA, SKU No. P8415-1KU) and 100 μM flg22, was added into each well. Luminescence was measured by a Photek camera system (Thermo, USA). Each data point represented eight biological replicates.

For the determination of defence-related gene expression, 10-day-old homozygous mutant and WT rice plants were treated with 1 μM flg22. After 1 h, total RNA was isolated from 100 mg rice roots using the RNA prep Pure Micro Kit (TianGen Biotech, Beijing, China). The expression levels of five defence-related genes, including 9bH-pimara-7,15-diene synthase enzyme (*OsKS4*, Os04g10060) (Shimizu et al., 2010), phenylalanine ammonia-lyase-like (*OsPAL4*, Os02g0627100) (Liang et al., 2022), enhanced disease susceptibility 1 protein (*OsEDS1*, Os09g22450) (Singh and Shah, 2012) and pathogenesis-related protein (*OsPR1a*, Os07g0418500; *OsPR4*, Os11g0592200) (Wang et al., 2006), were determined by qRT-PCR. These experiments were performed three times, with three technical replicates for each reaction. The roots of three plants under the same condition were pooled to generate one sample.

In order to check callose deposition, the roots of different plants were treated with flg22. The assay was carried out according to the method described by Chen et al. (2018), and the callose points were observed under UV light (340–380 nm, Nikon ECLIPSE Ni) and quantified with ImageJ software.

### Phenotypic observations

The WT rice and ‘transgene-free’ homozygous mutant rice were germinated on B5 medium. The plant height and root length were measured at 10 day after germination. Subsequently, plants were transferred and cultivated in sand and soil (3:1) at 27°C in a greenhouse. At the mature stage, agronomic traits were characterized by measuring the grain number per panicle, the seed setting rate and the thousand-grain weight of plants. Five plants were investigated for each line. Data are expressed as mean ± standard values. Statistical differences between treatments and controls were calculated by Student’s *t* test.

## Results

### CRISPR/Cas9-mediated targeted mutagenesis of *OsHPP04*

**Figure 1:**
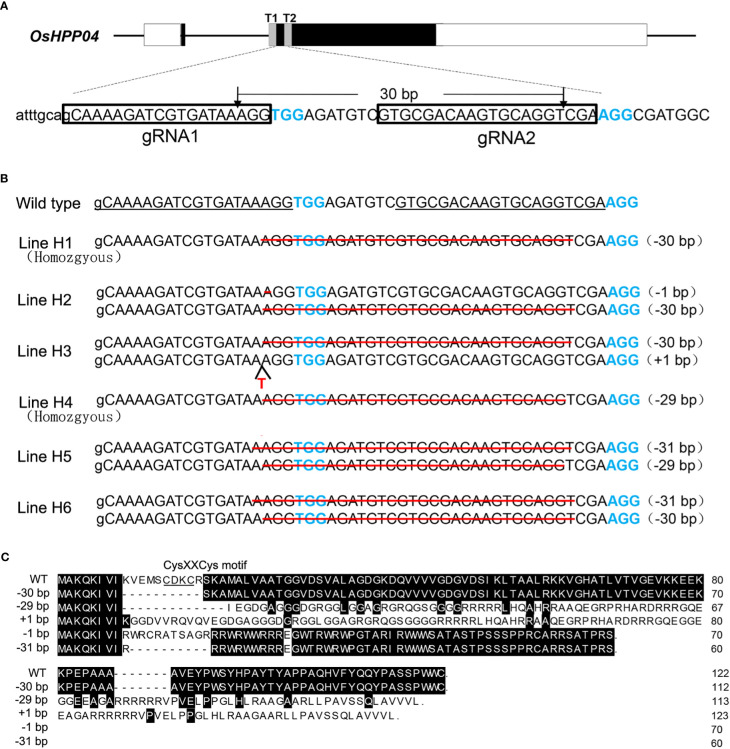
CRISPR/Cas9-targeted mutagenesis of *OsHPP04* in rice. **(A)** Target sites of CRISPR/Cas9. Two gRNAs (T1 and T2) were chosen in the second exon. Nucleotides in blue represent protospacer adjacent motif (PAM) sequences. **(B)** The mutated sequences of *OsHPP04* knockout rice lines. The number of base deletion and/or insertion is shown by the mark of minus (-) and plus (+) followed by a number in brackets, respectively. **(C)** Alignments of deduced *OsHPP04* amino acid sequence from *OsHPP04* knockout rice lines and wild-type rice (WT). Dots indicate the termination of translation.

Two 20-bp guide RNAs (gRNA1 and gRNA2) that are 30-bp apart in the second exon of the *OsHPP04* gene (LOC_Os02g37300) were used to construct the plant pYLCRISPR/Cas9-Pubi-H-*OsHPP04*-KO binary vector (**Figure 1A**), and the vector was introduced into the callus of Nipponbare rice by *Agrobacterium*-mediated transformation. Twenty transgenic events (T_0_ generation) were generated and validated due to the presence of *Cas9* code sequence by PCR (**Supplementary Figure 1**). Subsequently, the mutation type in each event was determined by amplifying and sequencing a 508-bp target region of *OsHPP04*. Decoding of sequencing showed six different mutant lines. They are two homozygous mutant lines (Line H1 of 30-bp deletion and Line H4 of 29-bp deletion) and four bi-allele mutant lines (Line H2 of 1-bp/30-bp deletion, Line H3 of 30-bp deletion/1-bp insertion, Line H5 of 31-bp/29-bp deletion and Line H6 of 31-bp/30-bp deletion) (**Figure 1B**). Among these, the mutations of 1-, 29-, and 31-bp deletion and 1-bp insertion cause the frameshift in the OsHPP04 coding region, generating the premature translation termination codon; whereas the 30-bp deletion doesn’t result in the frameshift mutation but destroys the metal-binding motif CysXXCys of OsHPP04 (**Figure 1C**).

Meanwhile, the potential off-target sites of the two gRNAs were predicted by CRISPR-P. Three most likely off-target sites of these two gRNAs in the six T_0_ plants were selected, amplified and sequenced. No mutations were observed in all potential off-target sites, indicating that the two gRNAs have a specificity for targeting the *OsHPP04* gene (**Table 1**).

**Table 1:** Mutations detected in the putative CRISPR/Cas9 off-target sites.

gRNA	Putative off-target gene	Off-target sequence*	No. of mismatches	Mutations
T1	Os03g0372600	GCAGAAGATCGTGATCAAGGTGG	2	not detected
	Os09g0240500	TCCGAAGATCGGGATAAAGGCGG	4	not detected
	Os06g0556300	TCCAAAGACCGCGATAAAGGTGG	4	not detected
T2	Os02g0584700	GAGCGACAAGTGCCGGTCGAAGG	2	not detected
	Os02g0585100	GAGCGACAAGTGCAGGCGGAAGG	3	not detected
	Os04g0585900	GTGCGACACGGGCAGGACGACGG	3	not detected

^*^Mismatching bases are in red. Nucleotides in blue represent protospacer adjacent motif (PAM) sequences.

### Identification of homozygous *OsHPP04* mutants in T_1_ generation

**Figure 2:**
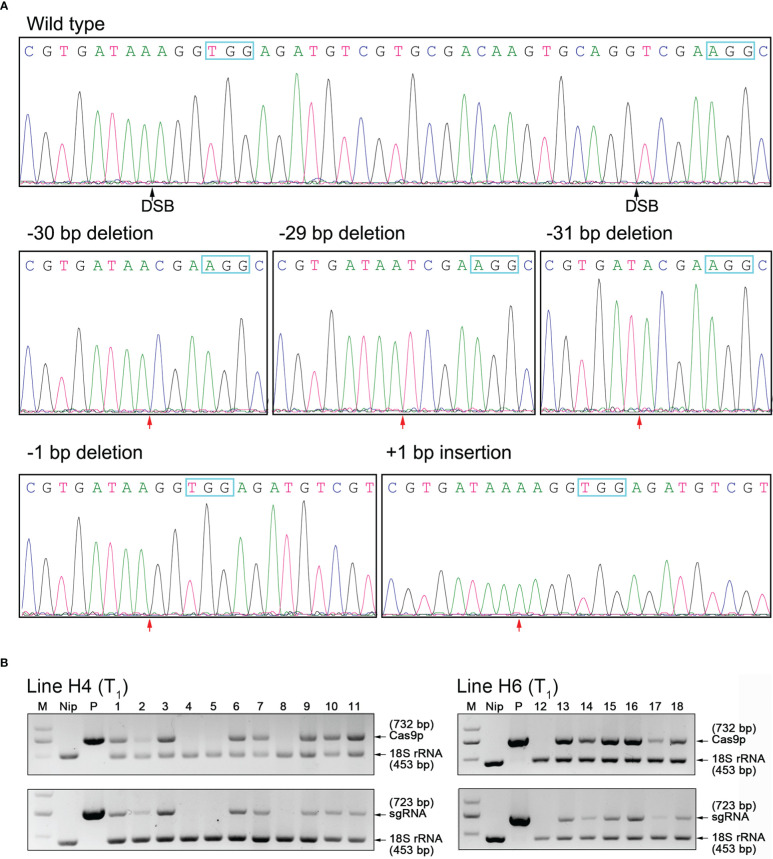
Detection of Homozygous *OsHPP04* mutants of rice. **(A)** Sanger sequencing results for *OsHPP04* mutants in T_1_ generation. The protospacer adjacent motif (PAM) sequences are shown in blue box. DSB indicate double-strand breaks. The red arrowheads indicate the location of mutations. **(B)** PCR-based test for identifying ‘transgene-free’ *OsHPP04* mutants of rice. Cas9p (732 bp), part of the *Cas9* coding sequence. sgRNA (723 bp), region ranging from the *OsU6a* promoter to the downstream of gRNA2. 18S rRNA (453 bp), part of the 18s rRNA sequence as a normalization control. M, DL2000 DNA marker. Nip, wild-type rice DNA. P, plant pYLCRISPR/Cas9-Pubi-H-*OsHPP04*-KO binary vector. Lanes 1-11 and 12-18, individual offspring of Line H4 and H6.

We further self-pollinated all the six T_0_ lines for more homozygous mutants with frameshift mutation types, and got five types of T_1_ generation homozygous mutant lines (**Figure 2A**). The ‘transgene-free’ plants without any transgenic elements of the *OsHPP04*-KO vectors were further identified from these edited events of T_1_ generation through a PCR strategy based on two sets of primer pairs amplifying *Cas9* and *gRNA* regions, respectively. Finally, three ‘transgene-free’ homozygous plants of 29-bp deletion and one ‘transgene-free’ homozygous plant of 31-bp deletion (**Figure 2B**) respectively from T_1_ segregants of Line H4 and Line H6 were obtained, named TF-H4-1 and TF-H6-1. No “transgene-free” plants from other lines were detected (**Supplementary Figure 2**). Other homozygous mutant lines containing transgenic elements were named as H1-1 (30-bp deletion), H2-1 (1-bp deletion), H3-1 (1-bp insertion), H5-1 (29-deletion) and H5-2 (31-bp deletion), respectively.

### Knockout of *OsHPP04* enhanced rice resistance to *M. graminicola*

**Figure 3:**
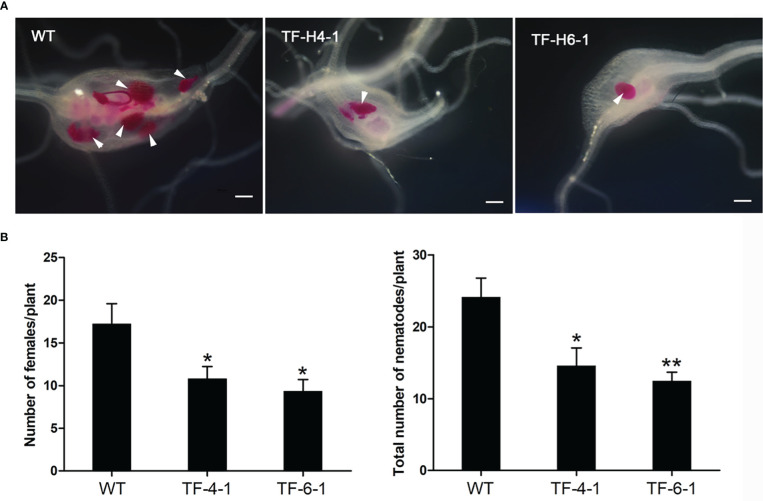
CRISPR/Cas9-mediated *OsHPP04* mutants exhibited enhanced rice resistance to *Meloidogyne graminicola*. **(A)** Representative pictures of galls in wild-type (WT) and two ‘transgene-free’ edited plants (TF-H4-1 and TF-H6-1) at 12 day post-inoculation (dpi) with *M. graminicola*. The white arrows indicate nematodes. Bars = 200 μm. **(B)** The number of adult females and nematodes in indicated rice lines at 12 dpi with *M. graminicola*. Data were analyzed by a two-tailed Student’s *t-test* (*P < 0.05, **P < 0.01. Values are mean ± *SE*).

We continued to self-pollinate T_1_ generation homozygous mutant lines to produce homozygous mutants of T_2_ generation, which were inoculated by *M. graminicola* for evaluating their resistance ability to nematodes. At 12 dpi, the number of nematodes was counted. In results, not only two ‘transgene-free’ mutant lines (TF-H4-1 and TF-H6-1) were significantly (*P*<0.05) more resistant to *M. graminicola*, as the average number of adult females and total nematodes was reduced by 37.4%-42.6% and 39.6%-41.9%, respectively (**Figure 3**), but also all homozygous mutant lines with transgenic elements (H1-1, H2-1, H3-1, H5-1 and H5-2) exhibited a higher resistance to *M. graminicola* (**Supplementary Figure 3**), compared to WT Nipponbare rice. These results demonstrated that CRISPR/Cas9-mediated mutagenesis of *OsHPP04* conferred robust resistance to *M. graminicola*.

### Loss of function of *OsHPP04* elevated plant immune responses in rice

**Figure 4:**
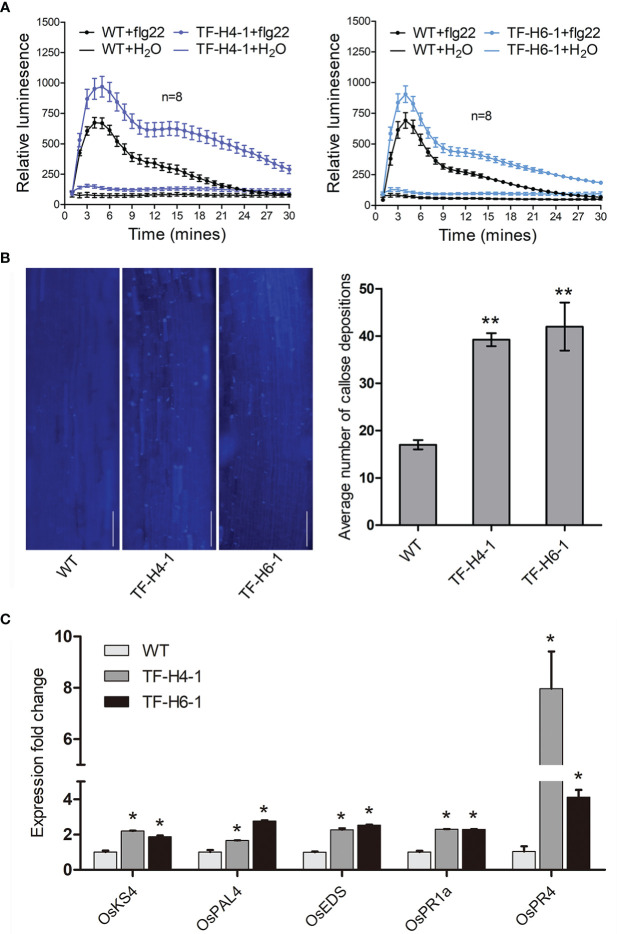
Plant immune responses in the loss-of-function mutants of *OsHPP04* after flg22 treatment. **(A)** ROS burst from the different shoot discs treated with 1 μM flg22 was measured by Photek camera system between 0 and 30 min. Bars represent mean ± *SE* (n = 8 biological replicates). **(B)** Callose deposition in rice roots after treatment with 1μM flg22 for 12 h. The average amount of callose depositions were measured by ImageJ. Data were analyzed by a two-tailed Student’s *t-test* (**P < 0.01. Values are mean ± *SE*). Bars = 50 μm. **(C)** Expression levels of defence-related genes (*OsKS4*, *OsPAL4*, *OsEDS1*, *OsPR1a* and *OsPR4*) were determined by quantitative real-time polymerase chain reaction in rice after treatment with 1 μM flg22 for 1 h. Values are mean ± *SE*, *P<0.05, Student’s *t*-test. The rice Ubiquitin gene (*OsUBQ*) was used as an internal control. WT: wild-type rice; TF-H4-1 and TF-H6-1: two ‘transgene-free’ *OsHPP04* mutant lines. Three independent experiments were performed with similar results.

Considering the *M. graminicola* effector MgMO289 plays a role in ROS scavenging to suppress plant immune responses by targeting OsHPP04 (Song et al., 2021), we hypothesized that OsHPP04 is a negative regulator of host immunity. Therefore, we investigated the flg22-induced PTI responses in the two *OsHPP04*-knockout lines TF-H4-1 and TF-H6-1. The results showed that the ROS level and callose deposition were obviously enhanced in these two *OsHPP04*-knockout lines compared with those in WT rice (**Figures 4A, B** **)**. Additionally, the differential expression of genes involved in plant innate immunity was investigated in rice roots using qRT-PCR. We selected several genes involved in the immune responses, such as *OsKS4*, *OsPAL4*, *OsEDS1*, *OsPR1a* and *OsPR4*. The results showed that the expression levels of these genes were significantly higher in these two mutant lines compared with those in WT rice (**Figure 4C**). Taken together, these results showed that OsHPP04 plays a negatively role in the plant basal defense.

### Main agronomic traits in *OsHPP04* mutants show no difference from wild-type rice

**Figure 5:**
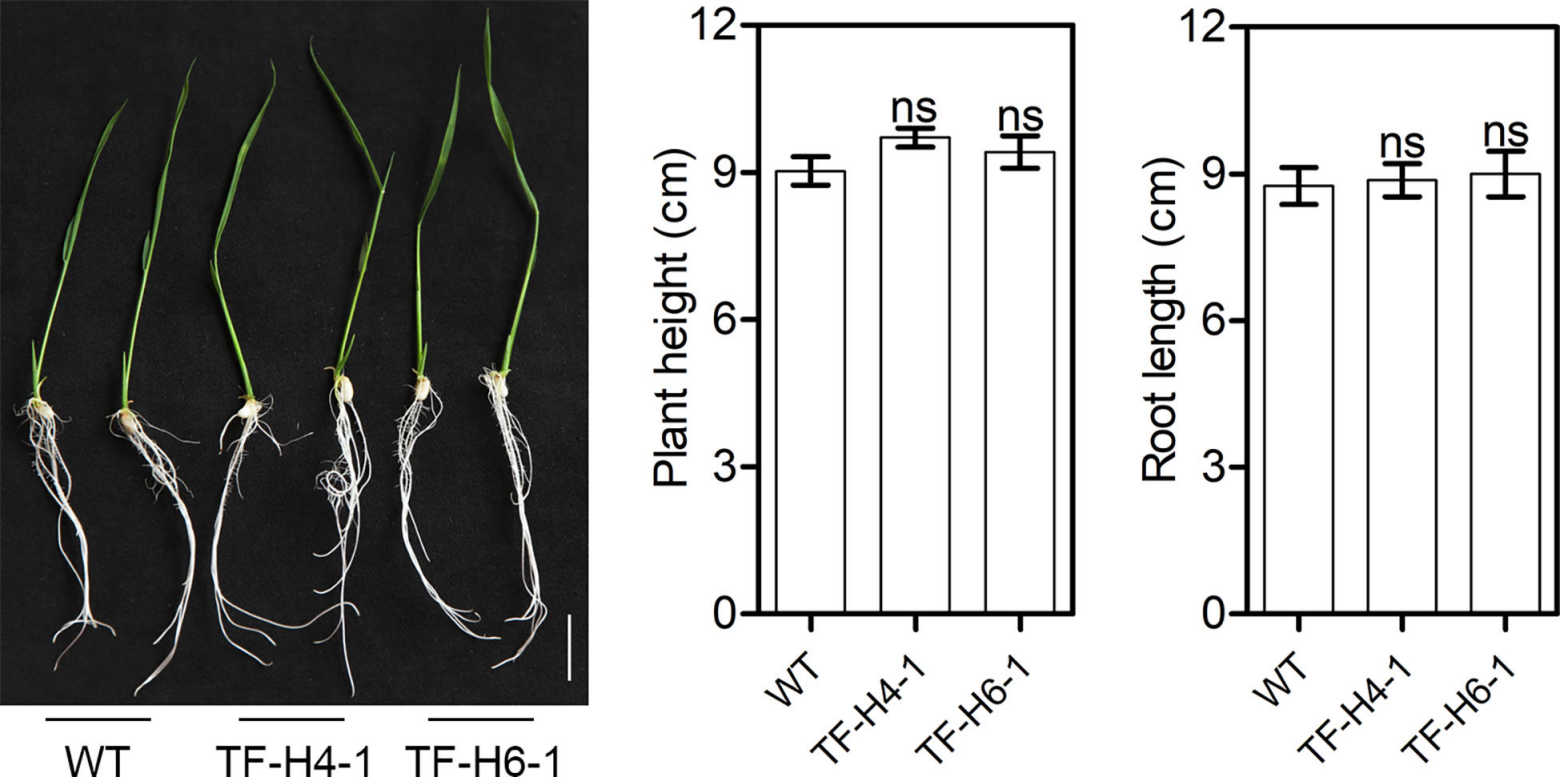
Growth phenotypes of ‘transgene-free’ *OsHPP04* knockout rice at 10 day after germination. Bars = 5 cm. Data were analyzed by a two-tailed Student’s *t-test* (ns *P* > 0.05. Values are mean ± *SE*). WT: wild-type rice; TF-H4-1 and TF-H6-1: two ‘transgene-free’ *OsHPP04* mutant lines.

**Figure 6:**
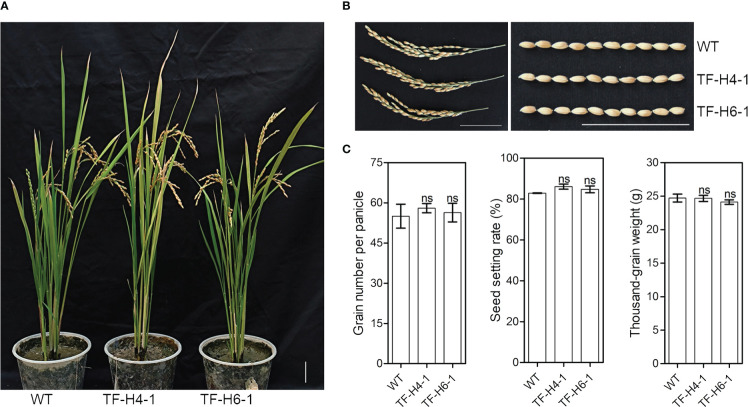
The major agronomic traits of ‘transgene-free’ *OsHPP04* knockout rice at mature stage. **(A)** The morphological phenotypes of rice. **(B)** Panicle and seeds. **(C)** The grain number per panicle, seed setting rate and thousand-grain weight. Data were analyzed by a two-tailed Student’s *t-test* (ns *P* > 0.05. Values are mean ± *SE*). WT: wild-type rice. TF-H4-1 and TF-H6-1: two ‘transgene-free’ *OsHPP04* mutant lines. Bars = 5 cm.

To know whether the edited rice have knockout of *OsHPP04* has negative effects on growth, we assessed the growth phenotypes and agronomic traits of the two ‘transgene-free’ mutant lines grown under greenhouse conditions. At 10 day after germination, the plant height and root length had no significant differences in mutants and WT controls (**Figure 5**). Furthermore, the plant architecture, panicle type, grain number per panicle, seed setting rate and thousand-grain weight of mature rice mutants were also not significantly different from those in WT controls (**Figure 6**). These results suggest that knockout of the *OsHPP04* do not cause any negative effects on rice growth.

## Discussion

The CRISPR/Cas9 technology has been widely used to develop mutant crops with resistance abilities to different pathogens including virus, fungus and bacterium by modifying *S* genes (Garcia-Ruiz, 2018; Liu et al., 2021; Koseoglou et al., 2022). Take rice for example, loss-function of the *S* gene *bsr-k1*, which encodes TPR-domain RNA-binding protein, led to broad-spectrum resistance against blast and blight in rice; mutation of the *S* gene *Eukaryotic translation initiation factor 4G* (*eIF4G*) in rice confer resistance to *Rice tungro spherical virus* (Macovei et al., 2018; Zhou et al., 2018). To our knowledge, phytopathogens usually exploit plants’ *S* genes to facilitate their invasion and proliferation dependent on three main molecular mechanisms: (i) assists in host recognition and penetration; (ii) negative regulators of host immune signaling; and (iii) contribute to pathogen proliferation and spread (van Schie and Takken, 2014). Many metallochaperones function in plant defence, moreover, several plant immune receptors have been found to carry HMA, implying that HMA probably participate in plant immunity (Sarris et al., 2016). Disrupting this kind of genes may interfere with the compatibility between hosts and pathogens. Our previous study showed that the rice copper metallochaperone *OsHPP04* might be an *S* gene for *M. graminicola*, and OsHPP04 was targeted by the *M. graminicola* effector MgMO289. Furthermore OsHPP04 interacted and activated the cytosolic COPPER/ZINC-SUPEROXIDE DISMUTASE 2 (cCu/Zn-SOD2) to decrease ROS and promote nematode parasitism (Song et al., 2021). In this study, plant immune responses, including the ROS level, defence-related genes expression level and callose deposition, were obviously enhanced in *OsHPP04*-knockout lines compared with those in WT rice. All these suggest that *OsHPP04* should be an *S* gene as a negative regulator of host immunity. Accordingly we edited the *OsHPP04* using the CRISPR/Cas9 technology and successfully increased *M. graminicola*-resistance in rice. This is the first report of using CRISPR/Cas9-mediated knockout of plant *S* genes to produce PPN resistant plant material.

In this study, we construct the plant pYLCRISPR/Cas9-Pubi-H-*OsHPP04*-KO binary vector to target the second exon of the *OsHPP04* gene. Finally we obtained the homozygous mutations of 1-, 29-, and 31-bp deletion and 1-bp insertion that cause the frameshift in the OsHPP04 coding region, generating the premature translation termination codon. Meanwhile, no mutations were observed in the examined potential off-target sites. As expected, all these OsHPP04-edited lines exhibit a significantly higher resistance to *M. graminicola* compared to the WT rice. Meantime, we also got a 30-bp deletion line without mutations in the examined potential off-target sites, which doesn’t result in the frameshift mutation. Interestingly, this line also has an obviously higher resistance to *M. graminicola*. Our previous study showed that the OsHPP04 contains one conserved metal-binding motif CysXXCys within a βαββαβ-fold HMA (Song et al., 2021), which defines protein function. The 30-bp deletion mutant destroys the metal-binding motif CysXXCys of OsHPP04. Usually the mutation of a few amino acids doesn’t affect protein function, however, many studies indicated that deletion of amino acids in key domains can disable protein function (Yang et al., 2021; de Figueiredo et al., 2022; Liu et al., 2022a). Therefore, the mutant line of 30-bp deletion (10 amino acids deletion) increased resistance against *M. graminicola* probably because of the 30-bp deletion which destroys the key domain of CysXXCys.

Previously, several *Arabidopsis S* genes of plant nematode disease, such as *KMD3*, *bHLH25/27*, *CCS52A1/B*, *FTRc* and *HIPP27*, have been reported, and knockout or knockdown of these *S* genes by T-DNA insertion or RNA interference enhanced *Arabidopsis* resistance to certain PPNs (van Schie and Takken, 2014; Lin et al., 2016; Radakovic et al., 2018). Although these methods could be effective for the introduction of PPN resistance into crops, transgenic plants are still opposed by many governments and people (Pyott et al., 2016). With advances in CRISPR/Cas9 gene editing technology, ‘transgene-free’ crops can be produced by selfing or backcrossing to the original parental line (Gao et al., 2016; Pyott et al., 2016). In the present study, two lines were indeed validated to be ‘transgene-free’ homozygous lines, i.e. non-transgenic mutants. Further research showed that the two ‘transgene-free’ homozygous mutant lines have no any adverse effects on plant growth. As is known to us, the mutants should be disease resistant with no fitness cost when using *S* genes for resistance breeding. However, *S* genes should not be considered a silver bullet, since *S*-gene-mediated resistance often accompanies several fitness costs, such as reduced growth, yield, and fertility. Hence, it is necessary to determine the main agronomic traits of the mutants (Li et al., 2020; Yang et al., 2020; Tao et al., 2021; Tripathi et al., 2021; Zhou et al., 2022; Liu et al., 2022b).

In summary, we successfully created genetically stable *OsHPP04* non-transgenic rice homozygous mutants, which confer enhanced resistance to the rice RKN and have no any adverse effects on plant growth, providing new insights into potential approaches for engineering resistance resources to PPN disease.

## Data availability statement

The original contributions presented in the study are included in the article/**Supplementary Material**. Further inquiries can be directed to the corresponding author.

## Author contributions

KZ, QH and BL planned and designed the research. QH, YC, YZ, HS, CH and TS performed experiments. KZ, QH, BL and CL analyzed the data. QH and BL wrote the manuscript. KZ and JL revised the manuscript. All authors contributed to the article and approved the submitted version.
