# Supplementary material for: CRISPR/Cas9-mediated mutagenesis of the susceptibility gene OsHPP04 in rice confers enhanced resistance to rice root-knot nematode
Source: Front Plant Sci. 2023 Mar 14;14:1134653. doi: 10.3389/fpls.2023.1134653 (PMC10043372; doi:10.3389/fpls.2023.1134653)
Supplement: Supplementary file 1 [file DataSheet_1.docx]

***Supplementary Material***

**Supplementary Table 1.** Primers used in this study.

| Primer | Sequence (5’→3’) | Purpose |
| --- | --- | --- |
| U-F | CTCCGTTTTACCTGTGGAATCG | CRISPR/Cas9 vector construction |
| gR-R | CGGAGGAAAATTCCATCCAC |  |
| gRT1 | CAAAAGATCGTGATAAAGGgttttagagctagaaat |  |
| OsU6aT1 | CCTTTATCACGATCTTTTGCggcagccaagccagca |  |
| gRT2 | TGCGACAAGTGCAGGTCGAgttttagagctagaaat |  |
| OsU6bT2 | TCGACCTGCACTTGTCGCACaacacaagcggcagc |  |
| Pps-R | TTCAGAggtctcTaccgACTAGTCACGCGTATGGAATCGGCAGCAAA |  |
| Pgs-2 | AGCGTGggtctcGtcagggTCCATCCACTCCAAGCTC |  |
| Pps-2 | TTCAGAggtctcTctgacacTGGAATCGGCAGCAAAGG |  |
| Pgs-L | AGCGTGggtctcGctcgACGCGTATCCATCCACTCCAAGC |  |
| OsHPP04-Pf | TATCTCACTCATCCACGCCA | PCR amplification of target region containing the *OsHPP04* target site |
| OsHPP04-Pr | CTTCTTGACCTCGCCGAC |  |
| OsHPP04-cx-F | CTGAGCTAATTCCCAAGC | Sanger sequencing |
| Cas9p-F | ACTGGAGGCAGCTTCTCAAC | PCR amplification of part of the Cas9 coding sequence |
| Cas9p-R | TAGCGATGAGCTTGTCCGAG |  |
| sgRNA-F | AACTTAATCGCCTTGCAGCAC | PCR amplification of region ranging from the OsU6a promoter to the downstream of gRNA2 |
| sgRNA-R | GGCTTAGTTCGTTCTTGCATCC |  |
| 18S rRNA-F | TACCGTCCTAGTCTCAACCA | PCR amplification of 18S rRNA fragment for normalization control |
| 18S rRNA-R | AGAACATCTAAGGGCATCACA |  |
| T1-off target-1-F | AAAGCTCGTATAATGACGCAGA | PCR amplification of DNA fragment covering each potential off-target site and sanger sequencing |
| T1-off target-1-R | CTTCACCCCTGCAAAATTAATCA |  |
| T1-off-target-1-cx-F | CATGTCGTCCAAGGTCAG |  |
| T1-off target-2-F | ACCAATCTTTTCTCCCTTATCCC |  |
| T1-off target-2-R | GAACATATCCGTTTGGTTAGCA |  |
| T1-off-target-2-cx-F | ATGGAATTACTTGATTCG |  |
| T1-off target-3-F | GTAGTCCACCTCTCTAGGGC |  |
| T1-off target-3-R | GAGATCGGATTGGTTTCTCTGA |  |
| T1-off-target-3-cx-F | CTGTCCATGCGTGGTACT |  |
| T2-off target-1-F | AGCAACACGATTCAGGCTTT |  |
| T2-off target-1-R | TTTGTTTAGAATATAGACCACGAGC |  |
| T2-off-target-1-cx-F | CATCAATGGCCAAGGTAC |  |
| T2-off target-2-F | ATAGGAGCTAATGCCAGGGG |  |
| T2-off target-2-R | GTAGTAGTAGTAGTACGGCGG |  |
| T2-off-target-2-cx-F | CGACCTCTTCGTTCATCC |  |
| T2-off target-3-F | CACGCAAAAGTTTGTTGAGG |  |
| T2-off target-3-R | CGCCTAGCTCCAATGTTCTAA |  |
| T2-off-target-3-cx-F | ATCGACGCCAACCCGTGG |  |
| OsKS4-qPCR-F | TCGCATTGCGTGTGCAA | qRT-PCR for rice defense marker gene expression |
| OsKS4-qPCR-R | TTGGAACTTCCGACATCGAAA |  |
| OsPAL4-qPCR-F | ATCACCGAGTGCAGGTCGTATC |  |
| OsPAL4-qPCR-R | TCGCCGGTCAGGTACTTTGTTC |  |
| OsEDS1-qPCR-F | GCTCCAGTCCGAGGTATCTA |  |
| OsEDS1-qPCR-R | GAGCCCCAAAGGTTACACAA |  |
| OsPR1a-qPCR-F | TCGTATGCTATGCTACGTGTTT |  |
| OsPR1a-qPCR-F | CACTAAGCAAATACGGCTGACA |  |
| OsPR4-qPCR-F | GGCAAGTGTATCCAGGTGAA |  |
| OsPR4-qPCR-R | ATGCAAAGAGGCATGACCAA |  |
| OsUBQ-qPCR-F | CCAGTAAGTCCTCAGCCATGGAG | Rice housekeeping gene (*OsUBQ*) used as qRT-PCR reference |
| OsUBQ-qPCR-R | GGACACAATGATTAGGGATC |  |


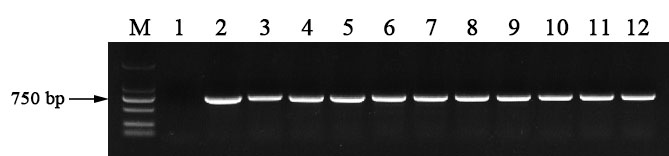


**Supplementary Figure 1.** PCR verification of *Cas9* in some T_0_ generation rice. M: DNA Marker DS2000; 1: Wild-type plant; 2: positive plasmid; 3-12: T_0_ generation transgenic plants.


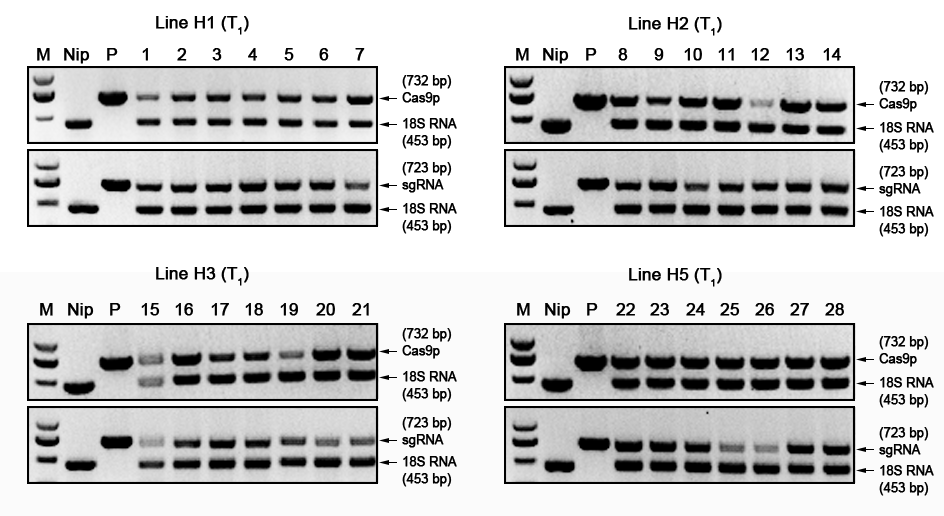


**Supplementary Figure** 2. PCR-based test for identifying ‘transgene-free’ *OsHPP04* mutants of rice. Cas9p (732 bp), part of the *Cas9* coding sequence. sgRNA (723 bp), region ranging from the *OsU6a* promoter to the downstream of gRNA2. 18S rRNA (453 bp), part of the 18s rRNA sequence as a normalization control. M, DL2000 DNA marker. Nip, wild-type rice DNA. P, plant pYLCRISPR/Cas9-Pubi-H-*OsHPP04*-KO binary vector. Lanes 1-7, Lanes 8-14, Lanes 15-21 and 22-28, individual offspring of Line H1, LineH2, Line H3 and H5, respectively.


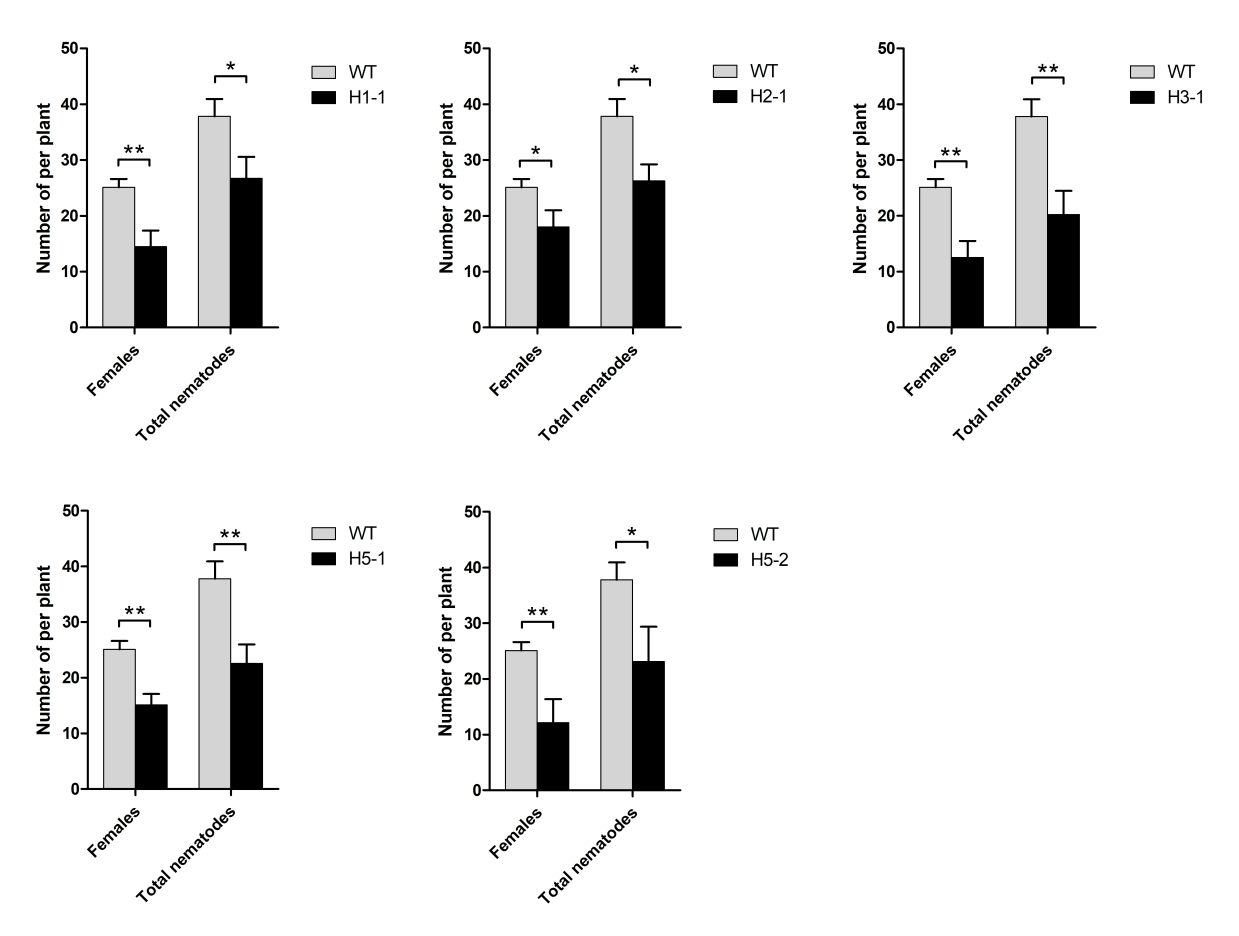


**Supplementary Figure 3.** The number of adult females and nematodes in homozygous mutant lines containing transgenic elements at 12 day post inoculation. WT: wild-type rice; H1-1: 30-bp deletion mutant line; H2-1: 1-bp deletion mutant line; H3-1: 1-bp insertion mutant line; H5-1: 29-bp deletion mutant line; H5-2: 31-bp deletion mutant line. Data were analyzed by a two-tailed Student’s *t-test* (* *P* < 0.05, ** *P* < 0.01. Values are mean ± *SE*).
